# Detecting PI3K and TP53 Pathway Disruptions in Early‐Onset Colorectal Cancer Among Hispanic/Latino Patients

**DOI:** 10.1002/cam4.70791

**Published:** 2025-04-01

**Authors:** Cecilia Monge, Brigette Waldrup, Sophia Manjarrez, Francisco G. Carranza, Enrique Velazquez‐Villarreal

**Affiliations:** ^1^ Center for Cancer Research, National Cancer Institute Bethesda Maryland USA; ^2^ Department of Integrative Translational Sciences City of Hope, Beckman Research Institute Duarte California USA; ^3^ City of Hope Comprehensive Cancer Center Duarte California USA

**Keywords:** cancer disparities, early‐onset colorectal cancer, genetic mutations, PI3K pathway, precision medicine, TP53 pathway

## Abstract

**Background/Objectives:**

This study aims to characterize PI3K and TP53 pathway alterations in Hispanic/Latino patients with early‐onset colorectal cancer (CRC), focusing on potential differences compared to non‐Hispanic White patients. Understanding these differences may shed light on the molecular basis of CRC health disparities.

**Methods:**

Using cBioPortal, we conducted a bioinformatics analysis to evaluate CRC mutations within the PI3K and TP53 pathways. CRC patients were stratified by age and ethnicity: (1) early‐onset (< 50 years) versus late‐onset (≥ 50 years) and (2) early‐onset in Hispanic/Latino patients compared to early‐onset in non‐Hispanic White patients. Mutation frequencies were assessed using descriptive statistics, with chi‐squared tests comparing proportions between early‐onset Hispanic/Latino and non‐Hispanic White groups. Kaplan–Meier survival curves were generated to assess overall survival for early‐onset Hispanic/Latino patients, stratified by the presence or absence of PI3K and TP53 pathway alterations.

**Results:**

Significant differences were noted when comparing early‐onset CRC in Hispanic/Latino patients to early‐onset CRC in non‐Hispanic White patients. PI3K (47.1% vs. 35.2%, *p* = 9.39e‐3) and TP53 (89.1% vs. 81.7%, *p* = 0.04) pathway alterations were more prevalent in early‐onset CRC among Hispanic/Latino patients, with AKT1 (5.1% vs. 1.8%, *p* = 0.03), INPP4B (4.3% vs. 1.4%, *p* = 0.04), and TSC1 (7.2% vs. 3.1% *p* = 0.03) gene alterations also significantly higher in this group. Significant differences were observed in TP53 mutations between colon adenocarcinomas (90% vs. 79.1%, *p* = 0.03), with higher prevalence in Hispanic/Latino patients when stratified by tumor site. No significant differences were observed between early‐onset and late‐onset CRC patients within the Hispanic/Latino cohort.

**Conclusions:**

These findings highlight the distinct role of PI3K and TP53 pathway disruptions in early‐onset CRC among Hispanic/Latino patients, suggesting that pathway‐specific mechanisms may drive cancer health disparities. Insights from this study could inform the potential development of precision medicine approaches and targeted therapies aimed at addressing these disparities.

## Graphical abstract

This study identifies distinct PI3K and TP53 pathway disruptions in early‐onset colorectal cancer among Hispanic/Latino patients, with findings suggesting a higher prevalence of PI3K alterations and potential differences in outcomes associated with TP53 disruptions compared to non‐Hispanic Whites. While these results provide valuable insights into potential molecular drivers of disparities, further research is needed to fully understand their clinical implications. These findings may suggest new directions for precision medicine approaches aimed at addressing cancer health disparities and improving equity in colorectal cancer care.

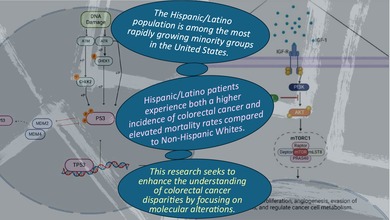

## 1 Introduction

Colorectal Cancer (CRC) is the third most prevalent cancer type and the second most common cancer‐related cause of death globally [1]. While the overall incidence of CRC has stabilized or decreased in higher‐income countries, a concerning trend has emerged, showing a rise in CRC among individuals under 50 years old [2, 3]. This disturbing trend is also accompanied by an increase in CRC‐related mortality [4]. This trend is further exacerbated when viewed through the lens of health disparities in the Hispanic/Latino population. The Hispanic/Latino population has the highest increase in early‐onset CRC incidence when compared to all other races in the United States [5]. The Hispanic/Latino population also has the highest increase in early‐onset CRC mortality when compared to their non‐Hispanic white counterparts [6, 7]. To effectively address this significant public health concern, several factors must be explored, including genomic determinants.

Early‐onset CRC is often diagnosed at more advanced stages, possibly because preventive screening measures typically do not begin until the age of 50 [8]. However, studies have emerged revealing early‐onset CRC has distinct molecular characteristics such as a significantly higher microsatellite instability distribution rate, elevated tumor mutation burden, and PD‐L1 expression [2, 9]. In addition, LINE‐1 hypomethylation has been suggested as a distinct molecular biomarker of early‐onset CRC [10]. Multiple signaling pathways are implicated in the development and progression of CRC. Two key pathways involved are the PI3K and TP53 pathways.

PI3K pathway is a primary signaling pathway that regulates processes, including cell metabolism, apoptosis, and proliferation [11]. The pathway is controlled by four principal types of receptors: receptor tyrosine kinases (RTKs), which sense growth factors; cytokine receptors, associated with immune responses and cell growth; G‐protein coupled receptors (GPCRs), linked to metabolic functions; and integrins, which play a role in sensing cell–cell and cell‐matrix adhesion. Each of these receptor types initiates signaling cascades that regulate essential cellular processes, such as survival, growth, and communication [12]. Hyperactivation of the PI3K pathway has been associated with tumor cell proliferation, cell invasion, and reduced apoptosis [13]. In CRC, the most common genetic alterations include IGF2 overexpression, PIK3CA mutations, and PTEN mutations or deletions, with these changes present in approximately 40% of malignant tumors [12]. Clinical data have shown advanced stages of CRC with mutations in this pathway do not respond well to anti‐EGFR therapy [14]. Other key genes altered in this pathway that contribute to CRC development include AKT1 mutations, which are associated with disease progression and resistance to therapy via various downstream targets [12, 15]. mTOR complex 1 (mTORC1), which is activated by fully phosphorylated AKT, is one of the key targets within this signaling pathway [12]. mTORC1 plays a crucial role in regulating many metabolic processes that contribute to tumor growth and drug resistance. One example is its role as a key contributor to drug resistance in treatments targeting PI3K inhibitors [15]. Although alterations in the PI3K/AKT pathway play a critical role in CRC development, these aberrations have not been well‐characterized in early‐onset CRC in the Hispanic/Latino population.

The TP53 pathway regulates a wide range of cellular functions, including cell cycle arrest, DNA repair, apoptosis, autophagy, and metabolism. The primary gene in the TP53 pathway is the transcription factor p53, which is located in the nucleus and cytoplasm and specifically binds to DNA to regulate a diverse set of genes. P53, also known as the guardian of the genome, plays a crucial role in maintaining genomic stability and coordinating DNA damage repair response mechanisms and promoting cell‐cycle arrest through p21 and repressing cyclin‐dependent kinases (CDKs) and cyclin B [16]. CRC exhibits one of the highest incidences of TP53 mutations, with approximately 74% of tumors harboring a TP53 mutation. Currently, numerous drugs are undergoing clinical trials aimed at validating their safety and efficacy for use in CRC treatment [17]. Additionally, gain‐of‐function missense mutations in TP53 have been shown to contribute to therapy resistance [18]. Although alterations in the TP53 pathway are crucial for CRC development, their specific role in early‐onset CRC, especially among Latino populations, remains poorly understood.

Recent studies have highlighted the role of genetic and epigenetic factors in CRC progression, including inflammation‐driven mechanisms and non‐coding RNA regulatory pathways. For example, vitexin‐mediated activation of the Vitamin D receptor (VDR) has been shown to mitigate inflammation‐driven CRC progression, emphasizing the role of tumor microenvironment interactions in CRC development [19]. Similarly, emerging research suggests that tRNA‐derived small RNAs, such as tsRNA‐GlyGCC, modulate oncogenic signaling pathways in CRC and contribute to chemoresistance by targeting SPIB within the JAK1/STAT6 pathway [20]. These findings underscore the complexity of molecular pathways involved in CRC progression and raise important questions about ethnic‐specific genomic alterations that may influence disease outcomes.

In this study, we aim to perform a comprehensive molecular analysis of the PI3K and TP53 signaling pathways in CRC within the Hispanic/Latino population, comparing early‐onset CRC patients with those diagnosed in Hispanic/Latino patients over the age of 50. We also investigate other molecular characteristics of early‐onset CRC, including tumor mutation burden and common driver oncogenes seen in CRC in Hispanic/Latino patients.

## 2 Materials and Methods

To conduct our analysis, we utilized clinical and genomic data from 20 CRC datasets accessed via the cBioPortal database. These datasets included studies classified under colorectal adenocarcinoma, colon adenocarcinoma, and rectal adenocarcinoma, as well as data from the GENIE BPC CRC v2.0‐public dataset. Two studies focusing on metastatic CRC were excluded to ensure the analysis was limited to primary tumor patients. Following dataset selection, we applied a series of filtering criteria to refine our sample pool. Patients were included if they were identified as Hispanic or Latino, Spanish, NOS; Hispanic, NOS; Latino, NOS; or those with a Mexican or Spanish surname. Additional criteria included restricting samples to primary tumors, including only colon adenocarcinoma, rectal adenocarcinoma, and colorectal adenocarcinoma, confirming histology as adenocarcinoma, NOS, and ensuring one sample per patient. This process resulted in three datasets meeting all criteria: TCGA PanCancer Atlas, MSK Nat Commun 2022, and GENIE BPC CRC, comprising 302 Hispanic/Latino CRC patients (138 early‐onset and 164 late‐onset patients). For Non‐Hispanic White patients, 3110 CRC patients (897 early‐onset and 2213 late‐onset patients) were included using the same inclusion criteria but applied within this specific racial and ethnic group (Tables 1 and 2). Age at diagnosis was extracted from individual clinical records within the GENIE database. This study represents one of the largest comprehensive characterizations of PI3K and TP53 pathway alterations in an underserved population, providing critical insights into the molecular disparities in early‐ and late‐onset CRC.

**TABLE 1:** Patient demographics and clinical characteristics of the Hispanic/Latino (H/L) and non‐Hispanic White (NHW) cohorts.

Clinical feature	H/L cohort *n* (%)	NHW cohort *n* (%)
Age onset and gender		
Early‐onset (< 50) male	83 (27.5%)	503 (16.2%)
Early‐onset (< 50) female	55 (18.2%)	394 (12.7%)
Late‐onset (≥ 50) male	93 (30.8%)	1209 (38.9%)
Late‐onset (≥ 50) female	71 (23.5%)	1004 (32.3%)
Stage at diagnosis		
Stage 1–3	98 (32.5%)	965 (31.0%)
Stage 4	132 (43.7%)	1131 (36.4%)
NA	72 (23.8%)	1014 (32.6%)
Ethnicity		
Spanish NOS; Hispanic NOS, Latino NOS	270 (89.4%)	0 (0.0%)
Mexican (includes Chicano)	28 (9.3%)	0 (0.0%)
Other Spanish/Hispanic	1 (0.3%)	0 (0.0%)
Spanish surname only	3 (1.0%)	0 (0.0%)
Non‐Spanish; non‐Hispanic	0 (0.0%)	3110 (100.0%)

**TABLE 2:** Ethnicity‐associated differences in clinical features between Hispanic and Latino (H/L) and non‐Hispanic White (NHW) cohorts.

Clinical feature	Early‐onset HL *n* (%)	Late‐onset HL *n* (%)	*p*	Early‐onset HL *n* (%)	Early‐onset NHW *n* (%)	*p*
Median diagnosis age (IQR)	41 (36–46)	61 (55–69)	**< 0.05**	41 (36–46)	42 (38–47)	**< 0.05**
Median mutation count^a^	7 (5–10)	8 (6–10)	> 0.05	7 (5–10)	7 (5–10)	> 0.05
AKT1 mutation						
Present	7 (5.1%)	3 (1.8%)	> 0.05	7 (5.1%)	16 (1.8%)	**< 0.05**
Absent	131 (94.9%)	161 (98.2%)		131 (94.9%)	881 (98.2%)	
INPP4B mutation						
Present	6 (4.3%)	2 (1.2%)	> 0.05	6 (4.3%)	13 (1.4%)	**< 0.05**
Absent	132 (95.7%)	162 (98.8%)		132 (95.7%)	884 (98.6%)	
TSC1 mutation						
Present	10 (7.2%)	6 (3.7%)	> 0.05	10 (7.2%)	28 (3.1%)	**< 0.05**
Absent	128 (92.8%)	158 (96.3%)		128 (92.8%)	869 (96.9%)	

*Note*: Bold text indicates statistically significant differences by age (Early‐Onset HL vs. Late‐Onset HL) and by ethnicity (Early‐Onset HL vs. Early‐Onset NHW) based on chi‐square analysis of categorical variables (*p* < 0.05).

^a^ LO HL: NA 1, EO NHW: NA 8.

Pathway alterations were defined according to previously established criteria [18]. To create our analysis cohorts, we categorized patients into early‐onset (under 50 years of age) and late‐onset (50 years or older) groups. Ethnicity‐based classification divided participants into Hispanic/Latino and Non‐Hispanic White groups. We further stratified these groups based on the presence or absence of PI3K and TP53 pathway alterations, enabling a detailed examination of the interactions between age, ethnicity, and these molecular changes. Table 3 presents the number of patients included in the analysis of early‐onset and late‐onset Hispanic/Latino CRC patients, with a total of 138 early‐onset Hispanic/Latino patients and 164 late‐onset Hispanic/Latino patients. This analysis evaluates the prevalence of PI3K and TP53 pathway alterations in early‐ and late‐onset CRC within the Hispanic/Latino population. Table 4 expands on these findings by comparing early‐onset Hispanic/Latino and early‐onset non‐Hispanic White CRC patients. This cohort includes 138 early‐onset Hispanic/Latino patients and 897 early‐onset Hispanic/Latino patients, allowing for a comparative assessment of pathway alterations across ethnic groups. By integrating these stratifications, our study provides one of the most comprehensive characterizations of PI3K and TP53 pathway disruptions in an underserved population, offering valuable insights into potential molecular disparities and their implications for precision medicine in CRC.

**TABLE 3:** Rates of PI3K and TP53 pathway alterations among early‐onset and late‐onset Hispanic/Latino CRC patients.

	Early‐onset HL *n* (%)	Late‐onset HL *n* (%)	*p*
PI3K alterations present	55 (39.9%)	77 (47.0%)	0.2619
PI3K alterations absent	83 (60.1%)	87 (53.0%)	
TP53 alterations present	123 (89.1%)	134 (81.7%)	0.1005
TP53 alterations absent	15 (10.9%)	30 (18.3%)	

**TABLE 4:** Rates of PI3K and TP53 pathway alterations among early‐onset Hispanic/Latino and Non‐Hispanic White (NHW) CRC Patients.

	Early‐onset H/L *n* (%)	Early‐onset NHW *n* (%)	*p*
PI3K alterations present	65 (47.1%)	316 (35.2%)	**0.009392**
PI3K alterations absent	73 (52.9%)	581 (64.8%)	
TP53 alterations present	123 (89.1%)	732 (81.6%)	**0.04031**
TP53 alterations absent	15 (10.9%)	165 (18.4%)	

*Note*: Bold text indicates statistically significant differences by ethnicity (Early‐Onset H/L vs. Early‐Onset NHW) based on chi‐square analysis of categorical variables (*p* < 0.05).

Statistical analysis included chi‐squared tests to evaluate the independence of categorical variables and identify potential associations between age, ethnicity, and pathway alterations. Additionally, we stratified samples by tumor location, distinguishing between colon and rectal cancers. This level of stratification facilitated a nuanced analysis of the interplay between age, ethnicity, and tumor location in relation to pathway disruptions, offering deeper insights into patient heterogeneity and potential implications for treatment responses.

Kaplan–Meier survival analysis was employed to assess overall survival, focusing on the impact of PI3K and TP53 pathway alterations. Survival curves were constructed to illustrate survival probabilities over time, with patients grouped by the presence or absence of pathway disruptions. The log‐rank test was utilized to determine statistically significant differences between survival curves. Median survival times were calculated, accompanied by 95% confidence intervals to convey the precision of these estimates. This comprehensive methodological approach provided an in‐depth understanding of how specific pathway alterations may affect patient outcomes in early‐ and late‐onset CRC patients within the Hispanic/Latino population.

## 3 Results

From three cBioPortal projects that reported ethnicity, we constructed our Hispanic/Latino cohort, which comprised 302 samples, while the non‐Hispanic White cohort included 3110 samples. In the Hispanic/Latino cohort, 45.7% of patients presented with early‐onset CRC (diagnosed before age 50), while 54.3% were diagnosed at age 50 or older (Table 1). Comparatively, in the non‐Hispanic White cohort, 28.8% had early‐onset CRC, with 71.2% being diagnosed at age 50 or older. The Hispanic/Latino cohort consisted of 58.3% male and 41.7% female patients, whereas the non‐Hispanic White cohort was composed of 55% male and 45% female patients. At the time of diagnosis, 32.5% of patients in the Hispanic/Latino cohort were at Stages 0, I, II, and III, while 43.7% were at Stage IV. In contrast, the non‐Hispanic White cohort had 31% of patients diagnosed at Stages 0, I, II, and III and 36.4% at Stage IV, with 32.6% of non‐Hispanic White patients recorded as NA for stage at diagnosis. All patients in the Hispanic/Latino cohort identified as Hispanic or Latino, which highlights the targeted demographic of the study.

The comparative analysis of clinical features between early‐onset and late‐onset Hispanic/Latino CRC patients, as well as between early‐onset Hispanic/Latino and early‐onset non‐Hispanic White patients, reveals notable distinctions (Table 2). The median age at diagnosis for early‐onset Hispanic/Latino patients was 41 years (IQR 36–46), which was significantly younger than the median age of 61 years (IQR 55–69) observed in late‐onset Hispanic/Latino patients (*p* < 0.05). Similarly, a significant difference was detected between early‐onset Hispanic/Latino (median 41 years) and early‐onset non‐Hispanic White patients (median 42 years, IQR 38–47) (*p* < 0.05).

Mutation analysis indicated a median mutation count of 7 in early‐onset Hispanic/Latino patients, while late‐onset Hispanic/Latino patients exhibited a slightly higher median of 8; however, this difference was not statistically significant (*p* > 0.05). Likewise, early‐onset non‐Hispanic White patients showed an identical median mutation count of 7 compared to early‐onset Hispanic/Latino patients, with no significant difference observed (*p* > 0.05).

**FIGURE 1:**
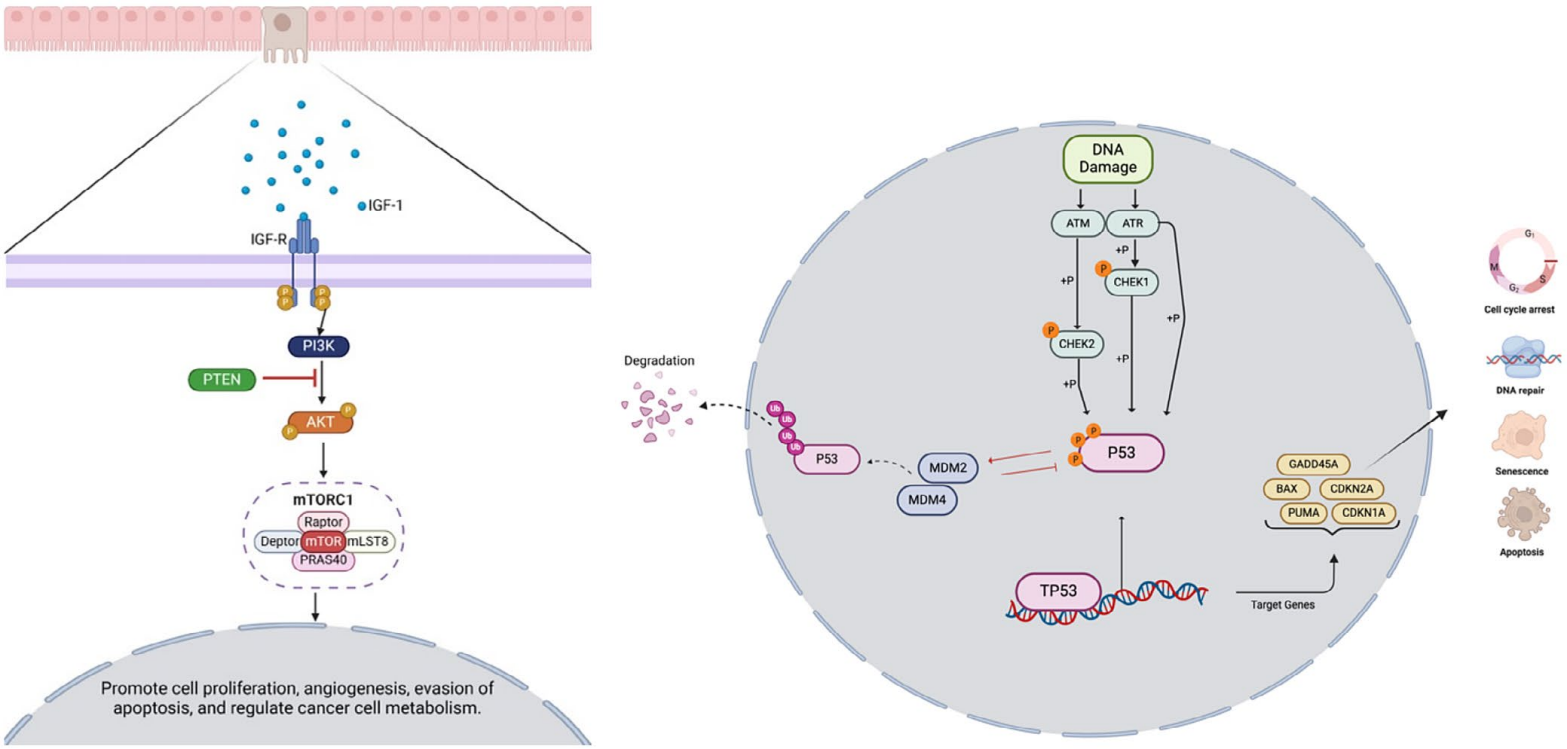
Illustration of the PI3K (left) and TP53, inside the nucleus, (right) signaling pathways [21, 22]. The left panel illustrates the PI3K pathway, a key intracellular signaling cascade involved in cell proliferation, survival, and metabolism. Upon activation by epidermal growth factor (EGF) binding to its receptor (EGFR), phosphorylation of PI3K occurs, leading to downstream activation of AKT. This process is negatively regulated by PTEN, a tumor suppressor that dephosphorylates PIP3 to inhibit pathway hyperactivation. Further downstream, mTORC1 is activated, contributing to cellular growth, metabolism, and cancer progression. Genetic alterations in components of this pathway, including PIK3CA mutations and PTEN deletions, are frequently observed in colorectal cancer (CRC). The right panel depicts the TP53 signaling pathway, a critical regulator of genomic stability and cellular response to DNA damage. DNA damage activates ATM and ATR kinases, which phosphorylate CHK1/CHK2, leading to the stabilization and activation of p53. Activated p53 transcriptionally regulates key target genes involved in cell cycle arrest (p21, CDKN1A), DNA repair, apoptosis (BAX, PUMA), and senescence. Negative regulation of p53 by MDM2 and MDM4 results in its degradation under normal conditions, while mutations in TP53 disrupt its tumor‐suppressive functions, contributing to CRC progression. These pathways play crucial roles in colorectal cancer development and therapy resistance, making them important targets for molecular characterization in early‐onset CRC among Hispanic/Latino patients.

**FIGURE 2:**
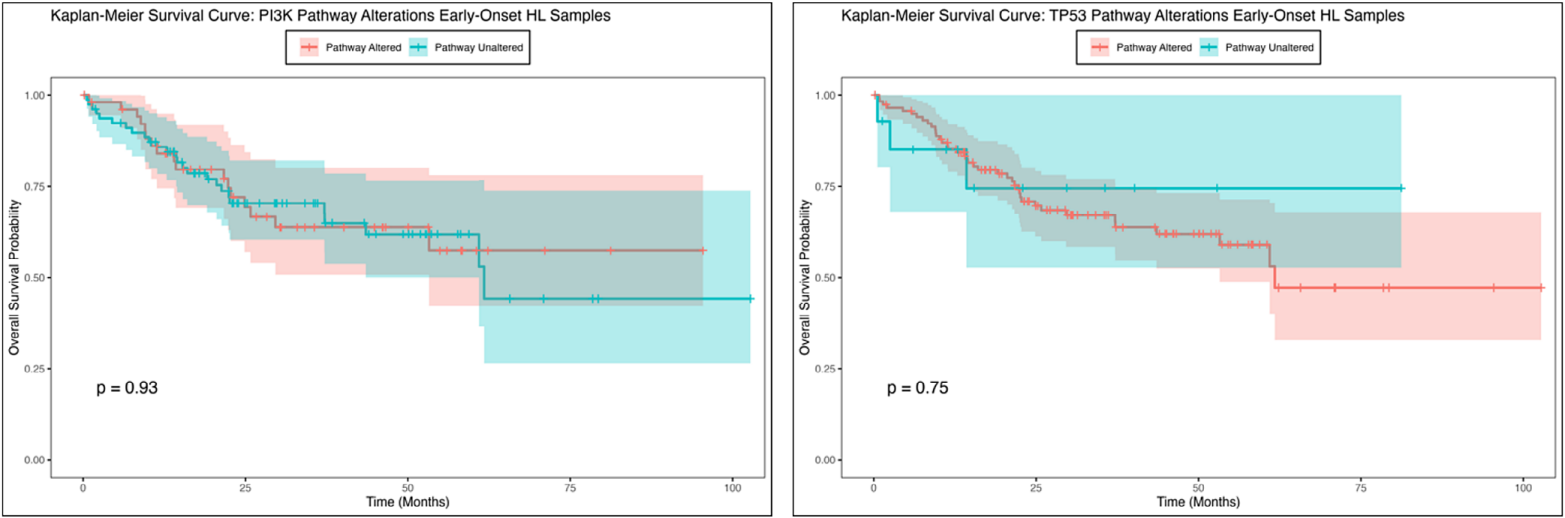
Overall survival curves of early‐onset Hispanic/Latino patients stratified by the presence or absence of PI3K (left) and TP53 (right) pathway alterations. The left panel illustrates the Kaplan–Meier survival curve for 132 early‐onset Hispanic/Latino CRC patients stratified by PI3K pathway alterations. Of these, 53 patients were in the altered group, while 79 patients were in the not altered group. Patients with PI3K pathway alterations (red curve) exhibited a trend toward reduced survival compared to those without alterations (blue curve), though the difference was not statistically significant (HR = 1.0, 95% CI: 0.56–1.9, *p* = 0.9). The right panel presents the Kaplan–Meier survival curve for early‐onset Hispanic/Latino CRC patients stratified by TP53 pathway alterations. Among these, 118 patients were in the altered group, while 14 patients were in the not altered group. Patients with TP53 pathway alterations (red curve) showed a slight trend toward worse survival outcomes compared to those without alterations (blue curve), though no significant survival difference was observed (HR = 0.82, 95% CI: 0.25–2.7, *p* = 0.7). Shaded regions around the curves represent 95% confidence intervals, and vertical tick marks indicate censored patients. These analyses highlight the potential impact of PI3K and TP53 pathway disruptions on patient survival in early‐onset CRC within the Hispanic/Latino population.

Furthermore, genes associated with the PI3K pathway, AKT1 (5.1% vs. 1.8%, *p* = 0.03), INPP4B (4.3% vs. 1.4%, *p* = 0.04), and TSC1 (7.2% vs. 3.1% *p* = 0.03) were found to be more prevalent in early‐onset Hispanic/Latino patients compared to non‐Hispanic White patients. These findings highlight potential genomic and clinical disparities between CRC subgroups that may have implications for understanding CRC pathogenesis and developing targeted interventions (Figures 1 and 2).

In our analysis of genetic alterations among Hispanic/Latino individuals with early‐onset and late‐onset CRC, we observed differences in the frequency of PI3K and TP53 pathway alterations (Table 3). PI3K alterations were present in 39.9% of early‐onset patients compared to 47% of late‐onset patients, although this difference did not reach statistical significance (*p* = 0.26). Conversely, the absence of PI3K alterations was more frequent in early‐onset patients (60.1%) than in late‐onset patients (53%). For TP53 alterations, there was a high prevalence in both groups, with 89.1% of early‐onset patients (123 out of 138) and 81.7% of late‐onset patients showing alterations, but without statistical significance (*p* = 0.1). This indicates that TP53 mutations are consistently common across both early‐onset and late‐onset patients. These findings suggest that PI3K and TP53 alterations may vary between early and late‐onset groups in Hispanic/Latino CRC patients.

In our analysis of genetic alterations in early‐onset CRC among Hispanic/Latino and Non‐Hispanic White individuals, we observed notable statistically significant differences in the frequency of PI3K and TP53 mutations. PI3K alterations were present in 47.1% of early‐onset Hispanic/Latino patients, compared to 35.2% of early‐onset non‐Hispanic White (*p* = 0.009). Conversely, the absence of PI3K alterations was more frequent among non‐Hispanic White individuals (64.8%) than among Hispanic/Latino individuals (52.9%). TP53 alterations, on the other hand, showed high prevalence in both early‐onset groups, with 89.1% of Hispanic/Latino patients and 81.6% of non‐Hispanic White patients carrying these mutations (*p* = 0.04). The absence of TP53 alterations was observed in 10.9% of Hispanic/Latino patients and 18.4% of non‐Hispanic White patients. These results highlight a potential difference in the genetic landscape between the two groups, with PI3K mutations being more common in early‐onset Hispanic/Latino individuals than in their non‐Hispanic White counterparts, while TP53 alterations were consistently high across both groups. Further statistical analyses are necessary to determine the significance of these findings and explore their potential implications for precision medicine and targeted treatment approaches in CRC.

The Kaplan–Meier survival analysis for early‐onset Hispanic/Latino CRC patients indicated no statistically significant difference in overall survival between those with and without PI3K pathway alterations (Figure 2). While the survival curves for patients with and without the genetic alteration appeared to diverge over time, hinting at a potential trend in survival outcomes, the *p*‐value (*p* = 0.93) suggested that this observed difference was not statistically significant. The confidence intervals surrounding each curve highlighted variability in the survival estimates at different time points, emphasizing the uncertainty of these findings. These results imply that, although survival differences may exist, the current data do not provide strong evidence of a significant impact of PI3K pathway alterations on overall survival within this sample. Further research involving larger cohorts or additional stratification is needed to clarify the role of genetic alterations in survival outcomes among early‐onset Hispanic/Latino CRC patients.

In contrast to the findings for PI3K pathway alterations, the investigation into the TP53 genetic pathway provided different insights. Although the Kaplan–Meier survival analysis between those with and without TP53 pathway alterations (Figure 2) revealed differences in early‐onset Hispanic/Latino CRC patients, the *p*‐value suggested that this difference was not statistically significant in overall survival. Patients with specific TP53 pathway genetic alterations showed a marked decline in survival probability early in the follow‐up period, with consistently lower survival rates compared to those without the alteration. Conversely, patients without the genetic alteration exhibited higher overall survival probabilities over time, with a more gradual decline in their survival curve. These findings suggest that while there is an apparent trend of poorer survival outcomes in patients with TP53 alterations, this difference may not be reliable and could be due to chance. Further studies with larger sample sizes would be necessary to confirm these findings.

Similar to the results for PI3K in the Hispanic/Latino cohort, the overall survival analysis for the non‐Hispanic White cohort (Figure S1) indicates that PI3K pathway alterations are significant determinants of survival outcomes in early‐onset CRC within this specific ethnic group. The *p*‐value of 2.5e‐4 indicates that this observed difference is statistically significant. In contrast, non‐Hispanic White patients with TP53 alterations did not exhibit a marked decline in survival compared to those without TP53 alterations, with a *p*‐value of 0.46, suggesting no statistically significant difference. This contrasts with the results seen in the Hispanic/Latino cohort, where TP53 alterations appeared to have a more pronounced impact on survival.

The alteration rates of PI3K and TP53 pathway‐related genes were analyzed among early‐onset and late‐onset Hispanic/Latino CRC patients to determine potential age‐related differences (Table S1). The analysis revealed that most genes associated with the PI3K pathway, including *PTEN*, *PIK3R1*, *PIK3R2*, and PIK3, displayed slightly higher alteration rates in early‐onset compared to late‐onset groups, although these differences were not statistically significant. An exception was *PIK3CA*, which showed alteration rates of 21.7% in early‐onset patients and 26.8% in late‐onset patients, with a *p*‐value of 0.37, indicating no statistically significant difference between the two groups. Similar to the PI3K pathway, most of the genes in the TP53 pathway displayed higher alteration rates in early‐onset compared to late‐onset patients. For example, TP53 showed a higher alteration rate in early‐onset patients (79.7%) compared to late‐onset patients (72.6%), though this difference was not statistically significant, with a *p*‐value of 0.19. Stratification by cancer type (e.g., colon vs. rectum adenocarcinoma) revealed no significant differences in PI3K and TP53 pathway alterations, indicating that these genetic variations remain consistent across CRC subtypes within this ethnic cohort (Table S2). These findings suggest that while alterations in PI3K pathway‐related genes do not show significant age‐related differences in early‐onset versus late‐onset Hispanic/Latino CRC patients, alterations in the TP53 pathway are more prevalent in early‐onset patients. However, the lack of statistical significance in both pathways highlights the complexity of these genetic alterations and their role in disease progression. These results underscore the need for further research to investigate the implications of these genetic differences in the context of precision medicine, especially for targeted treatment strategies in Hispanic/Latino CRC patients across various age groups.

Moreover, we compared the alteration rates of PI3K and TP53 pathway‐related genes between early‐onset Hispanic/Latino and non‐Hispanic White CRC patients (Table S3). The analysis identified three genes (AKT1, INPP4B, and TSC1) in the PI3K pathway that were statistically significantly more prevalent in early‐onset Hispanic/Latino CRC patients compared to non‐Hispanic White patients. When stratified by cancer type, early‐onset Hispanic/Latino patients with colon adenocarcinoma exhibited higher PI3K and TP53 pathway alterations compared to early‐onset non‐Hispanic White patients, with only the TP53 pathway alterations reaching statistical significance (*p* = 0.03). These results suggest that ethnic‐specific variations in the TP53 pathway are more pronounced in colon cancer, and TP53 pathway alterations may contribute to ethnic disparities in CRC prognosis and outcomes. These findings emphasize the importance of ethnicity‐specific studies to better understand the clinical implications and inform targeted treatment strategies for early‐onset CRC in diverse populations.

## 4 Discussion

The PI3K and TP53 signaling pathways are essential in the regulation of cellular functions, including cell growth, proliferation, and survival, and are frequently altered in CRC. The PI3K pathway, known for its role in cell metabolism and apoptosis, is often hyperactivated in CRC, contributing to tumorigenesis and resistance to targeted therapies [11, 12, 13]. The TP53 pathway, involving the tumor suppressor p53, regulates crucial processes such as DNA repair and cell cycle arrest and is mutated in approximately 74% of CRC patients, significantly impacting genomic stability [16, 17, 18]. While extensive research has characterized these pathways in the general population, few studies have explored their specific roles and disruption patterns in early‐onset CRC among Hispanic/Latino patients.

Our study aimed to address this knowledge gap by evaluating the alteration rates of PI3K and TP53 pathway‐related genes among early‐onset and late‐onset CRC patients within the Hispanic/Latino population and comparing these findings to early‐onset non‐Hispanic White patients. The results revealed significant differences in the genetic landscape of early‐onset Hispanic/Latino CRC patients compared to their non‐Hispanic White counterparts. Notably, PI3K pathway alterations were more prevalent in early‐onset Hispanic/Latino patients (47%) compared to non‐Hispanic White patients (35.2%, *p* = 0.009). Similarly, TP53 pathway alterations were more prevalent in early‐onset Hispanic/Latino patients (89.1%) than in non‐Hispanic White patients (81.6%, *p* = 0.04), highlighting significant ethnic disparities in the frequency of these genetic alterations. Specifically, three genes in the PI3K pathway showed significantly higher alteration rates in early‐onset Hispanic/Latino patients. These findings underscore the importance of understanding ethnicity‐specific genetic differences, as the PI3K pathway's hyperactivation could play a more prominent role in CRC pathogenesis in the Hispanic/Latino population, potentially influencing therapeutic responses and clinical outcomes.

Interestingly, our data showed no significant differences in PI3K pathway alterations when comparing early‐onset to late‐onset Hispanic/Latino CRC patients (39.9% vs. 47%, *p* = 0.2619), suggesting that PI3K‐related disruptions are not strongly age‐dependent in this ethnic group. Conversely, TP53 pathway alterations were consistently high in both early‐onset (89.1%) and late‐onset (81.7%) Hispanic/Latino patients (*p* = 0.1), reflecting the pathway's critical and widespread role in CRC regardless of age at diagnosis. These results align with the well‐documented role of TP53 mutations in CRC across various demographics [16, 17, 18], indicating that TP53 disruptions may be a common driver of CRC that transcends age and ethnicity.

Our findings provide compelling evidence of ethnic‐specific variations in PI3K and TP53 pathway alterations, which could have significant implications for CRC progression and treatment strategies [23, 24]. The higher prevalence of PI3K mutations in early‐onset Hispanic/Latino patients suggests potential differences in tumor biology that may influence disease aggressiveness, response to therapy, and overall prognosis. Notably, PI3K pathway alterations have been associated with resistance to anti‐EGFR therapy in CRC, which may have clinical relevance for treatment selection in Hispanic/Latino patients. In contrast, the significantly higher frequency of TP53 alterations in Hispanic/Latino patients compared to non‐Hispanic White patients underscores the potential role of TP53 mutations in CRC disparities. TP53 alterations are known to drive genomic instability, promote tumor progression, and contribute to therapy resistance, raising important questions about how these genetic disruptions impact survival outcomes and treatment responses in early‐onset CRC. These results highlight the need for precision medicine approaches tailored to underrepresented populations, particularly Hispanic/Latino patients who experience disproportionately rising rates of early‐onset CRC. Further studies integrating multi‐omics approaches, functional validation studies, and clinical outcomes data will be essential to determine how PI3K and TP53 alterations influence disease trajectories in diverse ethnic populations. Understanding these molecular differences may pave the way for targeted therapies that improve treatment efficacy and outcomes in early‐onset CRC among Hispanic/Latino patients.

Further, Kaplan–Meier survival analyses provided insights into the prognostic implications of these pathway alterations in early‐onset Hispanic/Latino CRC patients. The analysis revealed no significant impact of PI3K and TP53 pathway alterations on overall survival, despite observed divergence in survival curves over time. Interestingly, the presence of TP53 alterations was associated with poorer survival outcomes, reinforcing previous findings that highlight the tumor suppressor's role in mediating clinical prognosis [25, 26, 27]. The consistently high prevalence of TP53 mutations [28] in both early‐onset Hispanic/Latino patients (89.1%) and non‐Hispanic White patients (81.6%, *p* = 0.04), as well as PI3K pathway mutations in early‐onset Hispanic/Latino patients (47.1%) compared to non‐Hispanic White patients (35.2%, *p* = 0.009), indicates that TP53 and PI3K alterations may vary significantly between these ethnic groups; their impact on patient outcomes remains substantial.

Our stratified analysis by cancer type (e.g., colon vs. rectum adenocarcinoma) revealed significant differences in TP53 pathway alterations between early‐onset Hispanic/Latino and non‐Hispanic White patients, suggesting that ethnic‐specific variations may be pronounced for colon cancer. However, the observed disparities in PI3K pathway alterations between the ethnic groups highlight the importance of further investigating these molecular characteristics. The higher prevalence of PI3K pathway disruptions in the Hispanic/Latino population could point to unique underlying biological mechanisms influenced by genetic ancestry or environmental factors [29, 30, 31]. Studies have shown that genetic backgrounds can shape CRC development, with factors such as diet and lifestyle contributing to variations in mutation profiles and disease progression [30].

The implications of these findings are significant for the development of targeted therapies and precision medicine. The elevated rates of PI3K pathway alterations in early‐onset Hispanic/Latino patients may indicate that therapies targeting the PI3K/AKT/mTOR axis could be more effective in this population. Additionally, the consistent presence of TP53 mutations across both Hispanic/Latino and non‐Hispanic White groups underscores the need for novel strategies to target this pathway, as traditional therapeutic options remain limited [17]. High tumor mutation burden (TMB), which has been observed in early‐onset CRC patients, could also be leveraged to improve treatment responses, particularly in immunotherapy settings [11].

Our findings reveal a significant overrepresentation of PI3K and TP53 pathway alterations in early‐onset Hispanic/Latino CRC patients compared to their non‐Hispanic White counterparts, suggesting potential molecular mechanisms underlying CRC disparities in this population. These results align with emerging evidence emphasizing the role of ethnic‐specific variations in tumor biology and treatment response. Previous studies have shown that chronic inflammation plays a pivotal role in CRC pathogenesis, with VDR‐mediated macrophage polarization acting as a protective mechanism against inflammation‐driven tumor progression [19]. Given that PI3K pathway hyperactivation is linked to tumor proliferation and resistance to targeted therapy, it is plausible that these genomic alterations contribute to the aggressive clinical trajectory observed in early‐onset CRC among Hispanic/Latino patients. Additionally, the significant enrichment of TP53 alterations raises important questions regarding genomic instability and therapy resistance in this group. The role of non‐coding RNAs, such as tsRNA‐GlyGCC, in regulating CRC progression and chemoresistance through SPIB and the JAK1/STAT6 pathway further highlights the complexity of molecular interactions in CRC disparities [20]. Taken together, these insights emphasize the need for precision medicine strategies that account for ethnic‐specific genomic profiles in CRC treatment. Future research integrating multi‐omics approaches, tumor microenvironment analysis, and novel therapeutic targets will be essential to address the rising burden of early‐onset CRC in Hispanic/Latino patients and improve patient outcomes through targeted intervention strategies.

Despite the strengths of this study, several limitations must be acknowledged. The retrospective nature of bioinformatics analyses and the potential for selection bias inherent in publicly available genomic databases may affect the generalizability of these findings. Moreover, the underrepresentation of Hispanic/Latino patients in genomic databases poses challenges for comprehensive analyses of molecular disparities across diverse subpopulations [29, 32, 33, 34]. Larger, prospective studies are needed to confirm these findings and to explore the underlying biological mechanisms that contribute to the observed differences in pathway alterations between ethnic groups and age groups [21, 22, 35, 36].

## 5 Conclusions

In conclusion, our findings highlight the distinct role of PI3K and TP53 pathway disruptions in early‐onset CRC among Hispanic/Latino patients. The significantly higher alteration rates in the PI3K pathway and the consistent prevalence of TP53 mutations emphasize the need for ethnicity‐specific studies to better understand the clinical and biological implications of these pathways. Insights from this study may inform the development of precision medicine approaches aimed at reducing CRC health disparities and improving outcomes for diverse patient populations.

## Author Contributions

**Cecilia Monge:** writing – original draft, writing – review and editing, supervision, conceptualization, investigation. **Brigette Waldrup:** investigation, methodology, validation, visualization, formal analysis, data curation, software. **Sophia Manjarrez:** writing – review and editing. **Francisco G. Carranza:** writing – review and editing. **Enrique Velazquez‐Villarreal:** conceptualization, investigation, funding acquisition, writing – original draft, methodology, validation, visualization, writing – review and editing, software, formal analysis, project administration, data curation, supervision, resources.

## Ethics Statement

This study utilized publicly available data from the cBioPortal database, which aggregates de‐identified patient information from prior studies approved by the respective institutional review boards (IRBs). As no new patient data were collected or identifiable information analyzed, ethical approval was not required for this research.

## Consent

The authors have nothing to report.

## Conflicts of Interest

The authors declare no conflicts of interest.

## Supporting information


Figure S1.

Table S1.

Table S2.

Table S3.

Table S4.

## Data Availability

All data used in the present study is publicly available at https://www.cbioportal.org/ and https://genie.cbioportal.org. Additional data can be provided upon reasonable request to the authors.
