# Supplementary material for: Detecting PI3K and TP53 Pathway Disruptions in Early‐Onset Colorectal Cancer Among Hispanic/Latino Patients
Source: Cancer Med. 2025 Apr 1;14(7):e70791. doi: 10.1002/cam4.70791 (PMC11959147; doi:10.1002/cam4.70791)

**Figure S1.** Overall survival curves of early-onset Non-Hispanic White (NHW) patients stratified by the presence or absence of PI3K (left) and TP53 (right) pathway alterations.

The left panel displays the Kaplan-Meier survival curve for 897 early-onset NHW colorectal cancer (CRC) patients stratified by PI3K pathway alterations. Of these, 581 patients were in the altered group, while 316 patients were in the not altered group. Patients with PI3K pathway alterations (blue curve) exhibited significantly worse overall survival compared to those without alterations (red curve), with a statistically significant difference (HR = 1.6, 95% CI: 1.3–2.1, p < 0.001). The right panel presents the Kaplan-Meier survival curve for early-onset NHW CRC patients stratified by TP53 pathway alterations. Among these, 732 patients were in the altered group, while 165 patients were in the not altered group. Patients with TP53 pathway alterations (red curve) showed no significant difference in survival outcomes compared to those without alterations (blue curve), as indicated by HR = 1.1 (95% CI: 0.84–1.5, p = 0.4). Shaded regions around the curves represent 95% confidence intervals, and vertical tick marks indicate censored patients. These findings suggest that PI3K pathway alterations have a more pronounced impact on survival outcomes in early-onset NHW patients, whereas TP53 pathway alterations do not appear to significantly affect survival in this population.


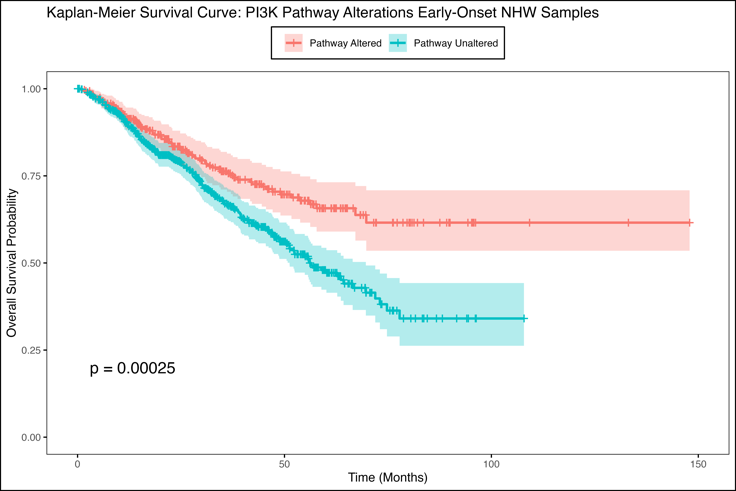


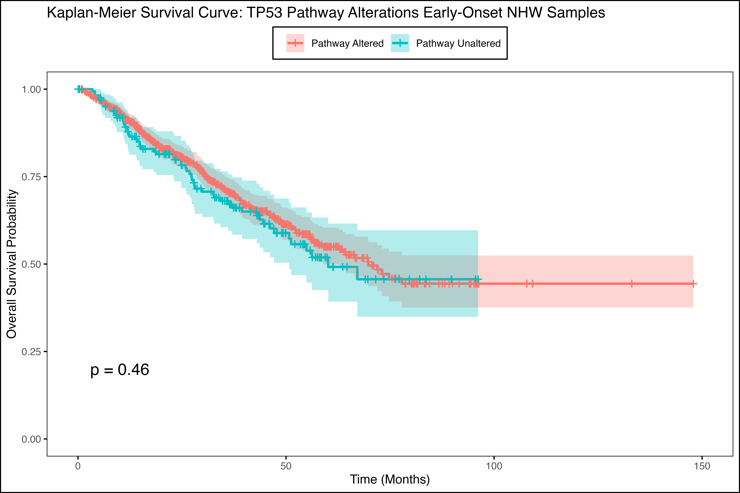

Supplement: Supplementary file 1 — Figure S1. [file CAM4-14-e70791-s001.docx]
