# Supplementary material for: Detecting PI3K and TP53 Pathway Disruptions in Early‐Onset Colorectal Cancer Among Hispanic/Latino Patients
Source: Cancer Med. 2025 Apr 1;14(7):e70791. doi: 10.1002/cam4.70791 (PMC11959147; doi:10.1002/cam4.70791)
Supplement: Supplementary file 2 — Table S1. [file CAM4-14-e70791-s005.docx]

| **Gene** | **Early Onset H/L n (%)** | **Late Onset H/L n (%)** | **p-value** |
| --- | --- | --- | --- |
| **IGF2 Mutation** | | | |
| Present | 1 (0.7%) | 2 (1.2%) | 1 |
| Absent | 137 (99.3%) | 162 (98.8%) |  |
| **IGF1R Mutation** | | | |
| Present | 6 (4.3%) | 5 (3.0%) | 0.557 |
| Absent | 132 (95.7%) | 159 (97.0%) |  |
| **INSR Mutation** | | | |
| Present | 4 (2.9%) | 7 (4.3%) | 0.7595 |
| Absent | 134 (97.1%) | 157 (95.7%) |  |
| **IRS1 Mutation** | | | |
| Present | 8 (5.8%) | 11 (6.7%) | 0.931 |
| Absent | 130 (94.2%) | 153 (93.3%) |  |
| **IRS2 Mutation** | | | |
| Present | 9 (6.5%) | 5 (3.0%) | 0.248 |
| Absent | 129 (93.5%) | 159 (97.0%) |  |
| **PIK3CA Mutation** | | | |
| Present | 30 (21.7%) | 44 (26.8%) | 0.3734 |
| Absent | 108 (78.3%) | 120 (73.2%) |  |
| **AKT1 Mutation** | | | |
| Present | 7 (5.1%) | 3 (1.8%) | 0.1948 |
| Absent | 131 (94.9%) | 161 (98.2%) |  |
| **AKT2 Mutation** | | | |
| Present | 1 (0.7%) | 2 (1.2%) | 1 |
| Absent | 137 (99.3%) | 162 (98.8%) |  |
| **MTOR Mutation** | | | |
| Present | 9 (6.5%) | 15 (9.1%) | 0.531 |
| Absent | 129 (93.5%) | 149 (90.9%) |  |
| **GRB10 Mutation** | | | |
| Present | 0 (0.0%) | 0 (0.0%) | 1 |
| Absent | 138 (100.0%) | 164 (100.0%) |  |
| **PTEN Mutation** | | | |
| Present | 12 (8.7%) | 11 (6.7%) | 0.6663 |
| Absent | 126 (91.3%) | 153 (93.3%) |  |
| **INPP4B Mutation** | | | |
| Present | 6 (4.3%) | 2 (1.2%) | 0.1483 |
| Absent | 132 (95.7%) | 162 (98.8%) |  |
| **PIK3R1 Mutation** | | | |
| Present | 6 (4.3%) | 6 (3.7%) | 0.7762 |
| Absent | 132 (95.7%) | 158 (96.3%) |  |
| **PIK3R2 Mutation** | | | |
| Present | 7 (5.1%) | 8 (4.9%) | 1 |
| Absent | 131 (94.9%) | 156 (95.1%) |  |
| **PIK3R3 Mutation** | | | |
| Present | 2 (1.4%) | 2 (1.2%) | 1 |
| Absent | 136 (98.6%) | 162 (98.8%) |  |
| **AKT3 Mutation** | | | |
| Present | 5 (3.6%) | 5 (3.0%) | 1 |
| Absent | 133 (96.4%) | 159 (97.0%) |  |
| **PPP2R1A Mutation** | | | |
| Present | 4 (2.9%) | 2 (1.2%) | 0.4174 |
| Absent | 134 (97.1%) | 162 (98.8%) |  |
| **TSC1 Mutation** | | | |
| Present | 10 (7.2%) | 6 (3.7%) | 0.259 |
| Absent | 128 (92.8%) | 158 (96.3%) |  |
| **TSC2 Mutation** | | | |
| Present | 10 (7.2%) | 12 (7.3%) | 1 |
| Absent | 128 (92.8%) | 152 (92.7%) |  |
| **STK11 Mutation** | | | |
| Present | 0 (0.0%) | 2 (1.2%) | 0.5021 |
| Absent | 138 (100.0%) | 162 (98.8%) |  |
| **RHEB Mutation** | | | |
| Present | 0 (0.0%) | 0 (0.0%) | 1 |
| Absent | 138 (100.0%) | 164 (100.0%) |  |
| **RICTOR Mutation** | | | |
| Present | 3 (2.2%) | 7 (4.3%) | 0.3541 |
| Absent | 135 (97.8%) | 157 (95.7%) |  |
| **RPTOR Mutation** | | | |
| Present | 7 (5.1%) | 3 (1.8%) | 0.2127 |
| Absent | 131 (94.9%) | 161 (98.2%) |  |

**Table S1.** Alteration Rates of PI3K and TP53 Pathway-Related Genes Among Early-Onset and Late-Onset Hispanic/Latino CRC Patients.

| **Gene** | **Early Onset H/L n (%)** | **Late Onset H/L n (%)** | **p-value** |
| --- | --- | --- | --- |
| **TP53 Mutation** | | | |
| Present | 110 (79.7%) | 119 (72.6%) | 0.19 |
| Absent | 28 (20.3%) | 45 (27.4%) |  |
| **MDM2 Mutation** | | | |
| Present | 0 (0.0%) | 2 (1.2%) | 0.5021 |
| Absent | 138 (100.0%) | 162 (98.8%) |  |
| **MDM4 Mutation** | | | |
| Present | 0 (0.0%) | 2 (1.2%) | 0.5021 |
| Absent | 138 (100.0%) | 162 (98.8%) |  |
| **CDKN1A Mutation** | | | |
| Present | 1 (0.7%) | 1 (0.6%) | 1 |
| Absent | 137 (99.3%) | 163 (99.4%) |  |
| **CDKN2A Mutation** | | | |
| Present | 2 (1.4%) | 2 (1.2%) | 1 |
| Absent | 136 (98.6%) | 162 (98.8%) |  |
| **ATM Mutation** | | | |
| Present | 16 (11.6%) | 17 (10.4%) | 0.8763 |
| Absent | 122 (88.4%) | 147 (89.6%) |  |
| **ATR Mutation** | | | |
| Present | 9 (6.5%) | 6 (3.7%) | 0.3816 |
| Absent | 129 (93.5%) | 158 (96.3%) |  |
| **CHEK1 Mutation** | | | |
| Present | 2 (1.4%) | 2 (1.2%) | 1 |
| Absent | 136 (98.6%) | 162 (98.8%) |  |
| **CHEK2 Mutation** | | | |
| Present | 3 (2.2%) | 5 (3.0%) | 0.7305 |
| Absent | 136 (98.6%) | 159 (97.0%) |  |
| **BAX Mutation** | | | |
| Present | 0 (0.0%) | 0 (0.0%) | 1 |
| Absent | 138 (100.0%) | 164 (100.0%) |  |
| **PUMA (BBC3) Mutation** | | | |
| Present | 0 (0.0%) | 1 (0.6%) | 1 |
| Absent | 138 (100.0%) | 163 (99.4%) |  |
| **GADD45A Mutation** | | | |
| Present | 0 (0.0%) | 0 (0.0%) | 1 |
| Absent | 138 (100.0%) | 164 (100.0%) |  |
| **PTEN Mutation** | | | |
| Present | 12 (8.7%) | 11 (6.7%) | 0.6663 |
| Absent | 126 (91.3%) | 153 (93.3%) |  |
