# Supplementary material for: Detecting PI3K and TP53 Pathway Disruptions in Early‐Onset Colorectal Cancer Among Hispanic/Latino Patients
Source: Cancer Med. 2025 Apr 1;14(7):e70791. doi: 10.1002/cam4.70791 (PMC11959147; doi:10.1002/cam4.70791)
Supplement: Supplementary file 3 — Table S2. [file CAM4-14-e70791-s002.docx]

**Table S2.** Rates of PI3K and TP53 pathway alterations in early-onset and late-onset Hispanic/Latino CRC patients, stratified by colon and rectal adenocarcinomas.

| **Colon Adenocarcinoma** | **Early-Onset HL n (%)** | **Late-Onset HL n (%)** | **p-value** |
| --- | --- | --- | --- |
| PI3K Alterations Present | 36 (45.0%) | 59 (53.6%) | 0.3037 |
| PI3K Alterations Absent | 44 (55.0%) | 51 (46.4%) |  |
| TP53 Alterations Present | 72 (90.0%) | 91 (82.7%) | 0.2274 |
| TP53 Alterations Absent | 8 (10.0%) | 19 (17.3%) |  |

| **Rectum Adenocarcinoma** | **Early-Onset HL n (%)** | **Late-Onset HL n (%)** | **p-value** |
| --- | --- | --- | --- |
| PI3K Alterations Present | 12 (30.0%) | 10 (32.3%) | 1 |
| PI3K Alterations Absent | 28 (70.0%) | 21 (67.7%) |  |
| TP53 Alterations Present | 36 (90.0%) | 26 (83.9%) | 0.4903 |
| TP53 Alterations Absent | 4 (10.0%) | 5 (16.1%) |  |
