# Supplementary material for: Detecting PI3K and TP53 Pathway Disruptions in Early‐Onset Colorectal Cancer Among Hispanic/Latino Patients
Source: Cancer Med. 2025 Apr 1;14(7):e70791. doi: 10.1002/cam4.70791 (PMC11959147; doi:10.1002/cam4.70791)
Supplement: Supplementary file 4 — Table S3. [file CAM4-14-e70791-s003.docx]

| **Gene** | **Early-Onset H/L n (%)** | **Early-Onset NHW n (%)** | **p-value** |
| --- | --- | --- | --- |
| **IGF2 Mutation** | | | |
| Present | 1 (0.7%) | 7 (0.8%) | 1 |
| Absent | 137 (99.3%) | 890 (99.2%) |  |
| **IGF1R Mutation** | | | |
| Present | 6 (4.3%) | 32 (3.6%) | 0.8331 |
| Absent | 132 (95.7%) | 865 (96.4%) |  |
| **INSR Mutation** | | | |
| Present | 4 (2.9%) | 18 (2.0%) | 0.521 |
| Absent | 134 (97.1%) | 879 (98.0%) |  |
| **IRS1 Mutation** | | | |
| Present | 8 (5.8%) | 41 (4.6%) | 0.6773 |
| Absent | 130 (94.2%) | 856 (95.4%) |  |
| **IRS2 Mutation** | | | |
| Present | 9 (6.5%) | 32 (3.6%) | 0.155 |
| Absent | 129 (93.5%) | 865 (96.4%) |  |
| **PIK3CA Mutation** | | | |
| Present | 30 (21.7%) | 185 (20.6%) | 0.851 |
| Absent | 108 (78.3%) | 712 (79.4%) |  |
| **AKT1 Mutation** | | | |
| Present | 7 (5.1%) | 16 (1.8%) | **0.03319** |
| Absent | 131 (94.9%) | 881 (98.2%) |  |
| **AKT2 Mutation** | | | |
| Present | 1 (0.7%) | 7 (0.8%) | 1 |
| Absent | 137 (99.3%) | 890 (99.2%) |  |
| **MTOR Mutation** | | | |
| Present | 9 (6.5%) | 46 (5.1%) | 0.6344 |
| Absent | 129 (93.5%) | 851 (94.9%) |  |
| **GRB10 Mutation** | | | |
| Present | 0 (0.0%) | 0 (0.0%) | 1 |
| Absent | 138 (100.0%) | 897 (100.0%) |  |
| **PTEN Mutation** | | | |
| Present | 12 (8.7%) | 61 (6.8%) | 0.5281 |
| Absent | 126 (91.3%) | 836 (93.2%) |  |
| **INPP4B Mutation** | | | |
| Present | 6 (4.3%) | 13 (1.4%) | **0.0433** |
| Absent | 132 (95.7%) | 884 (98.6%) |  |
| **PIK3R1 Mutation** | | | |
| Present | 6 (4.3%) | 55 (6.1%) | 0.526 |
| Absent | 132 (95.7%) | 842 (93.9%) |  |
| **PIK3R2 Mutation** | | | |
| Present | 7 (5.1%) | 18 (2.0%) | 0.05929 |
| Absent | 131 (94.9%) | 879 (98.0%) |  |
| **PIK3R3 Mutation** | | | |
| Present | 2 (1.4%) | 12 (1.3%) | 1 |
| Absent | 136 (98.6%) | 885 (98.7%) |  |
| **AKT3 Mutation** | | | |
| Present | 5 (3.6%) | 14 (1.6%) | 0.1804 |
| Absent | 133 (96.4%) | 883 (98.4%) |  |
| **PPP2R1A Mutation** | | | |
| Present | 4 (2.9%) | 24 (2.7%) | 0.781 |
| Absent | 134 (97.1%) | 873 (97.3%) |  |
| **TSC1 Mutation** | | | |
| Present | 10 (7.2%) | 28 (3.1%) | **0.03112** |
| Absent | 128 (92.8%) | 869 (96.9%) |  |
| **TSC2 Mutation** | | | |
| Present | 10 (7.2%) | 34 (3.8%) | 0.09962 |
| Absent | 128 (92.8%) | 863 (96.2%) |  |
| **STK11 Mutation** | | | |
| Present | 0 (0.0%) | 10 (1.1%) | 0.3751 |
| Absent | 138 (100.0%) | 887 (98.9%) |  |
| **RHEB Mutation** | | | |
| Present | 0 (0.0%) | 5 (0.6%) | 1 |
| Absent | 138 (100.0%) | 892 (99.4%) |  |
| **RICTOR Mutation** | | | |
| Present | 3 (2.2%) | 24 (2.7%) | 1 |
| Absent | 135 (97.8%) | 873 (97.3%) |  |
| **RPTOR Mutation** | | | |
| Present | 7 (5.1%) | 24 (2.7%) | 0.2042 |
| Absent | 131 (94.9%) | 873 (97.3%) |  |

**Table S3.** Alteration Rates of PI3K and TP53 Pathway-Related Genes in Early-Onset Hispanic/Latino and Non-Hispanic White CRC Patients.

| **Gene** | **Early Onset H/L n (%)** | **Early Onset NHW n (%)** | **p-value** |
| --- | --- | --- | --- |
| **TP53 Mutation** | | | |
| Present | 110 (79.7%) | 683 (76.1%) | 0.4158 |
| Absent | 28 (20.3%) | 214 (23.9%) |  |
| **MDM2 Mutation** | | | |
| Present | 0 (0.0%) | 8 (0.9%) | 0.6068 |
| Absent | 138 (100.0%) | 889 (99.1%) |  |
| **MDM4 Mutation** | | | |
| Present | 0 (0.0%) | 7 (0.8%) | 0.6031 |
| Absent | 138 (100.0%) | 890 (99.2%) |  |
| **CDKN1A Mutation** | | | |
| Present | 1 (0.7%) | 4 (0.4%) | 0.5118 |
| Absent | 137 (99.3%) | 893 (99.6%) |  |
| **CDKN2A Mutation** | | | |
| Present | 2 (1.4%) | 12 (1.3%) | 1 |
| Absent | 136 (98.6%) | 885 (98.7%) |  |
| **ATM Mutation** | | | |
| Present | 16 (11.6%) | 64 (7.1%) | 0.09794 |
| Absent | 122 (88.4%) | 833 (92.9%) |  |
| **ATR Mutation** | | | |
| Present | 9 (6.5%) | 44 (4.9%) | 0.5521 |
| Absent | 129 (93.5%) | 853 (95.1%) |  |
| **CHEK1 Mutation** | | | |
| Present | 2 (1.4%) | 12 (1.3%) | 1 |
| Absent | 136 (98.6%) | 885 (98.7%) |  |
| **CHEK2 Mutation** | | | |
| Present | 3 (2.2%) | 15 (1.7%) | 0.7234 |
| Absent | 136 (98.6%) | 882 (98.3%) |  |
| **BAX Mutation** | | | |
| Present | 0 (0.0%) | 0 (0.0%) | 1 |
| Absent | 138 (100.0%) | 897 (100.0%) |  |
| **PUMA (BBC3) Mutation** | | | |
| Present | 0 (0.0%) | 4 (0.4%) | 1 |
| Absent | 138 (100.0%) | 893 (99.6%) |  |
| **GADD45A Mutation** | | | |
| Present | 0 (0.0%) | 0 (0.0%) | 1 |
| Absent | 138 (100.0%) | 897 (100.0%) |  |
| **PTEN Mutation** | | | |
| Present | 12 (8.7%) | 61 (6.8%) | 0.5281 |
| Absent | 126 (91.3%) | 836 (93.2%) |  |
