# Supplementary material for: Detecting PI3K and TP53 Pathway Disruptions in Early‐Onset Colorectal Cancer Among Hispanic/Latino Patients
Source: Cancer Med. 2025 Apr 1;14(7):e70791. doi: 10.1002/cam4.70791 (PMC11959147; doi:10.1002/cam4.70791)
Supplement: Supplementary file 5 — Table S4. [file CAM4-14-e70791-s004.docx]

**Table S4.** Rates of PI3K and TP53 pathway alterations in early-onset Hispanic/Latino and Non-Hispanic White (NHW) CRC patients, stratified by colon and rectal adenocarcinomas.

| **Colon Adenocarcinoma** | **Early-Onset H/L n (%)** | **Early-Onset NHW n (%)** | **p-value** |
| --- | --- | --- | --- |
| PI3K Alterations Present | 36 (45.0%) | 194 (40.9%) | 0.5748 |
| PI3K Alterations Absent | 44 (55.0%) | 280 (59.1%) |  |
| TP53 Alterations Present | 72 (90.0%) | 375 (79.1%) | **0.03331** |
| TP53 Alterations Absent | 8 (10.0%) | 99 (20.9%) |  |

| **Rectum Adenocarcinoma** | **Early-Onset H/L n (%)** | **Early-Onset NHW n (%)** | **p-value** |
| --- | --- | --- | --- |
| PI3K Alterations Present | 12 (30.0%) | 84 (27.6%) | 0.8994 |
| PI3K Alterations Absent | 28 (70.0%) | 220 (72.4%) |  |
| TP53 Alterations Present | 36 (90.0%) | 259 (85.2%) | 0.6292 |
| TP53 Alterations Absent | 4 (10.0%) | 45 (14.8%) |  |
